# Potential of Plant Bioactive Compounds as SARS-CoV-2 Main Protease (M^pro^) and Spike (S) Glycoprotein Inhibitors: A Molecular Docking Study

**DOI:** 10.1155/2020/6307457

**Published:** 2020-12-23

**Authors:** Trina Ekawati Tallei, Sefren Geiner Tumilaar, Nurdjannah Jane Niode, Fatimawali, Billy Johnson Kepel, Rinaldi Idroes, Yunus Effendi, Shahenur Alam Sakib, Talha Bin Emran

**Affiliations:** ^1^Department of Biology, Faculty of Mathematics and Natural Sciences, Sam Ratulangi University, Manado 95115, Indonesia; ^2^Pharmacy Study Program, Faculty of Mathematics and Natural Sciences, Sam Ratulangi University, Manado 95115, Indonesia; ^3^Department of Dermatology and Venereology, Faculty of Medicine, University of Sam Ratulangi, Manado 95115, Indonesia; ^4^Department of Chemistry, Faculty of Medicine, Sam Ratulangi University, Manado 95115, Indonesia; ^5^Department of Pharmacy, Faculty of Mathematics and Natural Sciences, Syiah Kuala University, Banda Aceh 23111, Indonesia; ^6^Department of Biology, Faculty of Mathematics and Natural Sciences, Al Azhar University, South Jakarta 12110, Indonesia; ^7^Department of Theoretical and Computational Chemistry, University of Dhaka, Dhaka 1000, Bangladesh; ^8^Department of Pharmacy, BGC Trust University Bangladesh, Chittagong 4381, Bangladesh

## Abstract

Since the outbreak of the COVID-19 (coronavirus disease 19) pandemic, researchers have been trying to investigate several active compounds found in plants that have the potential to inhibit the proliferation of SARS-CoV-2 (severe acute respiratory syndrome coronavirus 2). The present study aimed to evaluate bioactive compounds found in plants using a molecular docking approach to inhibit the main protease (M^pro^) and spike (S) glycoprotein of SARS-CoV-2. The evaluation was performed on the docking scores calculated using AutoDock Vina (AV) as a docking engine. A rule of five (Ro5) was calculated to determine whether a compound meets the criteria as an active drug orally in humans. The determination of the docking score was performed by selecting the best conformation of the protein-ligand complex that had the highest affinity (most negative Gibbs' free energy of binding/Δ*G*). As a comparison, nelfinavir (an antiretroviral drug), chloroquine, and hydroxychloroquine sulfate (antimalarial drugs recommended by the FDA as emergency drugs) were used. The results showed that hesperidin, nabiximols, pectolinarin, epigallocatechin gallate, and rhoifolin had better poses than nelfinavir, chloroquine, and hydroxychloroquine sulfate as spike glycoprotein inhibitors. Hesperidin, rhoifolin, pectolinarin, and nabiximols had about the same pose as nelfinavir but were better than chloroquine and hydroxychloroquine sulfate as M^pro^ inhibitors. This finding implied that several natural compounds of plants evaluated in this study showed better binding free energy compared to nelfinavir, chloroquine, and hydroxychloroquine sulfate, which so far are recommended in the treatment of COVID-19. From quantum chemical DFT calculations, the ascending order of chemical reactivity of selected compounds was pectolinarin > hesperidin > rhoifolin > morin > epigallocatechin gallate. All isolated compounds' C=O regions are preferable for an electrophilic attack, and O-H regions are suitable for a nucleophilic attack. Furthermore, Homo-Lumo and global descriptor values indicated a satisfactory remarkable profile for the selected compounds. As judged by the RO5 and previous study by others, the compounds kaempferol, herbacetin, eugenol, and 6-shogaol have good oral bioavailability, so they are also seen as promising candidates for the development of drugs to treat infections caused by SARS-CoV-2. The present study identified plant-based compounds that can be further investigated in vitro and in vivo as lead compounds against SARS-CoV-2.

## 1. Introduction

Coronavirus disease 2019 (COVID-19) is a disease caused by a new type of transmissible pathogenic human severe acute respiratory syndrome coronavirus 2 (SARS-CoV-2), a member of *Betacoronaviruse* (Beta-CoVs) [1, 2]. As of 11 March 2020, the WHO has stated that COVID-19 has been characterized as a pandemic. The World Health Organization (2020), as of 3 April 2020, reported 932,166 confirmed cases and 46,764 deaths in 206 countries [3], while in Indonesia, the death toll of COVID-19 reached 6,150 with the number of positive cases of 137,468 people as of 15 August 2020, and patients who have recovered reached 91,321 [4].

COVID-19 infection is characterized by acute respiratory distress symptoms such as fever 38.1oC–39oC, dry cough, and shortness of breath with an incubation period of about five days (average 2–14 days) [5]. Until now, there is no specific therapy or vaccine available to treat and prevent COVID-19 [3, 6]. Therefore, there has been an increase in demand for the availability of medicines, vaccines, diagnostics, and reagents, all related to COVID-19. This phenomenon can lead to opportunities for irresponsible people to distribute falsified medical products.

Several agents are being used in clinical trials and protocols based on in vitro activity against SARS-CoV-2 or related viruses with limited clinical experience; however, the effectiveness of therapy for any type of drug has not been established [7]. Xu et al. [8] examined the effectiveness of tocilizumab (atlizumab, an immunosuppressive drug) in a retrospective analysis with the results such as reduced fever, oxygen demand, radiological features, and decreased C-reactive protein (CRP). Bian et al. [9], in an open-labeled clinical trial (concurrent controlled add-on clinical trial) of meplazumab, found a median virus clearance time, discharge time, and better repair time. In a study based on molecular dynamics simulation (MDS) of a docked protein-ligand compound, nelfinavir was predicted to be a COVID-19 drug candidate as the best potential inhibitor against main protease (M^pro^) [8]. On the other hand, despite little evidence on chloroquine and hydroxychloroquine's effectivity, these two antimalarial agents have been approved by the Food and Drug Administration (FDA) for emergency coronavirus treatment [6].

Because COVID-19 is a new disease with global severe health problems, research is still needed, including finding specific therapeutic regimens to overcome morbidity and mortality. The plant is one of the medicinal active compound sources that have been widely used to treat diseases caused by microbes [10–14]. There are many plant bioactive compounds reported to have activities as antifungal [15], antibacterial [16–18], and antiviral [19, 20]. The natural products that have been reported to have antiviral activity can be used as a starting point in finding potential bioactive compound candidates against SARS-CoV-2. Molecular docking can be used to predict how protein (receptor) interacts with bioactive compounds (ligands) [21, 22]. Several previous studies have been performed to investigate bioactive compounds in plants that have the potential to inhibit the proliferation of viruses [23–25].

Given the importance of early screening for the potential of bioactive compounds to find drug candidates or prevention of viral infections, this study aimed to evaluate several bioactive compounds found in several plants known by the community with a molecular docking approach. The study results are expected to be one of the references for further research in finding specific regimens to overcome COVID-19.

## 2. Materials and Methods

### 2.1. Determination of Ligands

The selection of plant-derived compounds used as ligands in the docking process in this study was based on in silico and in vitro experiments that we and other researchers have previously conducted on the antiviral activity of these compounds. The information was obtained through digital library search. These compounds were quinine [26], nabiximols (a combination of cannabidiol [27]and tetrahydrocannabinol [28]), hesperidin [29, 30], rhoifolin [31], pectolinarin [31], morin [32], epigallocatechin gallate [33, 34], herbacetin [31], ethyl cholate [35], kaempferol [36], tangeretin [37], chalcone [38], nobiletin [39], bis (3, 5, 5-trimethylhexyl) phthalate [35], 6-gingerol [40, 41], 6-shogaol [42], hydroxychloroquine sulfate [43], myristicin [44], and eugenol [45].

### 2.2. Determination of Receptors

Two SARS-CoV-2 proteins were chosen as drug discovery targets: main protease (M^pro^) (also called 3C-like protease- 3CL^pro^) (PDB code: 6LU7) and spike glycoprotein (S) (PDB code: 6VXX).

### 2.3. Ligand and Receptor Preparation

Three-dimensional (3D) structures of M^pro^ of SARS-CoV-2 were retrieved from the Protein Data Bank (http://www.rcsb.org//pdb) in pdb formats. These proteins were served as receptors in the docking process. The files were opened using BIOVIA Discovery Studio Visualizer 2020. Water molecules and ligands that were still attached to the receptors were removed, and the receptors were stored in the pdb format. Using Autodock Tools, polar hydrogen atoms were added to the receptors. Subsequently, the files were saved in the pdbqt format.

Ligand structures were obtained from the PubChem site (http://pubchem.ncbi.nlm.nih.gov). The search was performed by entering the name of the ligand in the search option. Each ligand's file was downloaded and saved. Files in the sdf format were converted to pdb using Open Babel. The pdb format of the ligand was opened using Autodock Tools. Torque adjustment was made by detecting root and adjusting as desired. The file was saved in the pdbqt format. Properties of active compounds were calculated using Lipinski's rule of five calculated on the SWISSADME predictor (http://www.swissadme.ch/) [46].

### 2.4. Active-Site Determination

The amino acids' location as active sites in the receptor region where the ligand was docked was determined using Autodock Tools. For this reason, a three-dimensional map of the grid box was made in the receptor region. The determination of this map was based on the type of docking used. A three-dimensional map was made as wide as the size of the receptor (spike glycoprotein) itself so that the ligand was likely to be docked to all parts of the receptor (blind docking). In M^pro^/3CL^pro^ docking, the three-dimensional map was of only the area's size to be docked (targeted docking).

### 2.5. Validation of Target Protein-Ligand Complex Structures

Validation was carried out by redocking the native ligand on the target protein, where the native ligand was first separated from the receptor using BIOVIA Discovery Studio Visualizer 2020. In this case, the receptor M^pro^ (PDB ID: 6LU7) was docked to cocrystallized native ligand inhibitor N3 N-[(5-methylisoxazol-3-yl) carbonyl] alanyl-L-valyl-N∼1∼-((1R, 2Z)-4-(benzyloxy)-4-oxo-1-{[(3R)-2-oxopyrrolidin-3-yl]methyl} but-2-enyl)-L-leucinamide [47]. The docking results will show the compound with the lowest bond energy when it binds to the target protein, to obtain the RMSD (root-mean-square distance) value of the docking compound. The method is said to be valid if the RMSD value obtained is ≤2 Å, so that docking of the test compound can be carried out with the target protein in the same grid box area [48, 49].

### 2.6. Receptor-Ligand Docking

The docking was performed using Autodock Vina (AV). Ligands and receptors that had been saved in the pdbqt format were copied into the Vina folder. Then, the Vina configuration file was typed into notepad, saved with the name “conf.txt.” Vina program was run through the command prompt.

### 2.7. Analysis and Visualization

The results of the docking calculation were shown in the output in notepad format. The ligands' docking conformation was determined by selecting the pose with the highest affinity (most negative Gibbs' free energy of binding/Δ*G*).

### 2.8. Computational DFT Method

The theoretical quantum chemical calculations were performed by mean Gaussian 09 Program (Revision E.01) [50] via gauss view 6.0.10 molecular visualization software program [51] on a Pentium IV/3.02 Hz personal computer (4 GB RAM), with Windows (10.0 version) platform. The *ab initio* theory was used to optimize the geometry using a DFT/6-31G basis set [52] and employing Becke's (B) [53] exchange functional combining Lee, Yang, and Parr's (LYP) correlation functional [54]. The electronic properties, such as optimized energies, point group, dipole moment, *E*_HOMO_, *E*_LUMO_, HOMO-LUMO energy gap, molecular electrostatic potential, and global reactivity descriptors, were calculated using the DFT/B3LYP method, based on the optimized structure in the gas phase.

## 3. Results

### 3.1. Rule of Five

Lipinski's rule of five (Ro5) of the docking compounds calculated on the SWISSADME predictor is shown in Table 1. Most of the compounds used in this study do not violate the Ro5. However, hesperidin, nabiximols, pectolinarin, epigallocatechin gallate, and rhoifolin do not meet the Ro5.

### 3.2. Molecular Docking

**Figure 1:**
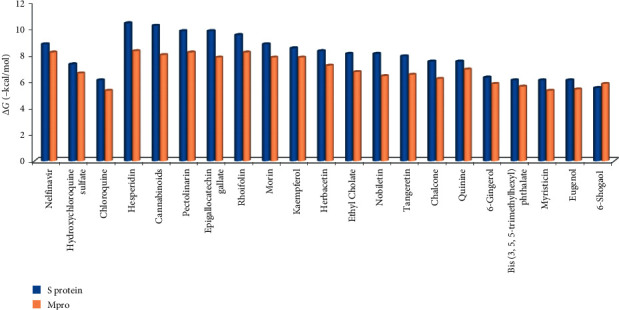
Histogram showing the binding energy value Δ*G* (−kcal/mol) of S protein and M^pro^ with several inhibitor compound candidates.

The estimation of free energy of binding between potential inhibitors and receptors was performed using a docking experiment.  Table 2 and Figure 1 show the docking analysis results between the selected compounds with M^pro^ (3CL^pro^) and S protein. The docking results showed that some compounds from plants with better binding positions with S protein than nelfinavir were hesperidin, nabiximols, pectolinarin, epigallocatechin gallate, and rhoifolin. Other compounds tended to be better positioned compared to chloroquine and hydroxychloroquine sulfate, except for 6-shogaol. Binding poses to M^pro^ that were better or equivalent to nelfinavir were hesperidin, rhoifolin, and pectolinarin. Some compounds showed better binding poses than chloroquine and hydroxychloroquine on M^pro^.

### 3.3. Docking Validation

**Figure 2:**
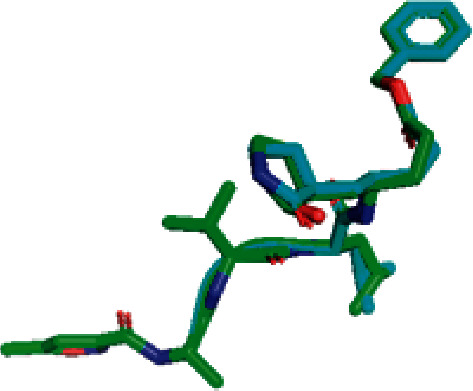
The result of superimposing the ligand position based on the redocking process of N3 with crystallography (green: crystallography result; blue: redocking result).

**Figure 3:**
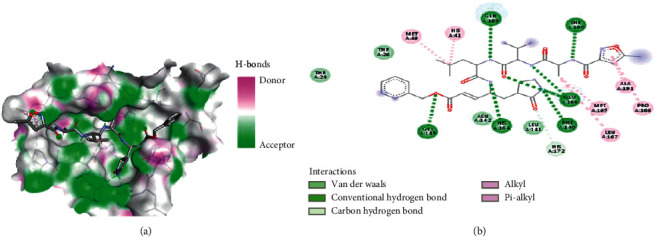
(a) The AV output, which shows the interacting amino acid residues of the main protease (M^pro^) with the native ligand. (b) 2D diagram showing the types of contacts formed between the receptor and ligand.

To evaluate whether the docking values can be accounted for, validation was carried out by redocking the M^pro^ receptor without ligands and with ligands that had previously been separated. The validation results are presented in Figures 2 and 3.

### 3.4. Visualization of the Docking Results

**Figure 4:**
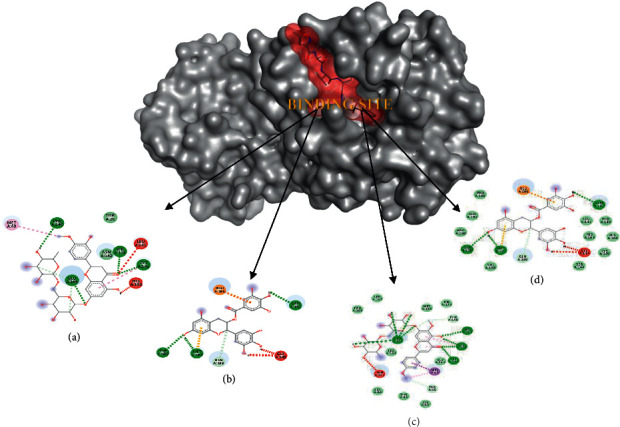
Docking position of hesperidin (a), nabiximols (b), pectolinarin (c), and epigallocatechin gallate (d) on M^pro^ protein.

**Figure 5:**
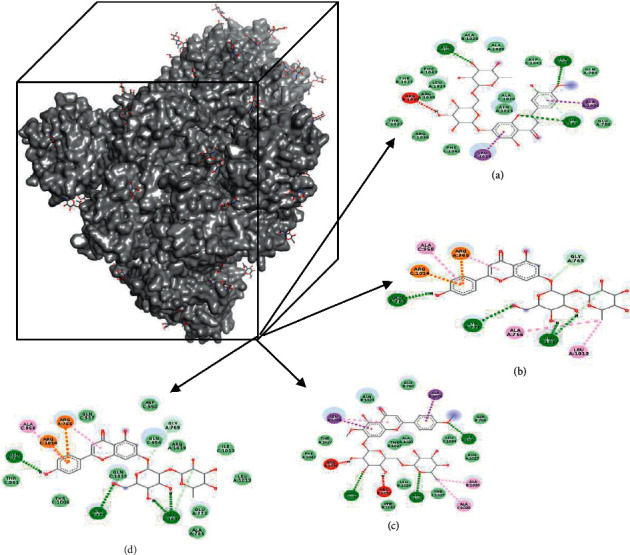
Docking position of hesperidin (a), nabiximols (b), pectolinarin (c), and epigallocatechin gallate (d) on spike protein.

Binding position on M^pro^ was evaluated and compared based on the native ligand. The result showed that four active compounds have different affinities to the receptor, but they bound specifically on the binding site (Figure 4). It is suggested that the ligand inhibited the activity of M^pro^. These data were also supported by molecular interaction analysis which revealed the specific interaction between ligands and M^pro^ (Table 1.) Interaction between active compounds and its receptor are mainly stabilized by the hydrogen bond and hydrophobic interaction. Four lead compound candidates showed the best poses with M^pro^ and spike protein, namely, hesperidin, nabiximols, pectolinarin, and epigallocatechin gallate. The binding site on spike protein was also evaluated in detail (Figure 5).

Detailed interaction was evaluated to show the complexes were stabilized by many types of interaction (Table 2). The docking process on spike protein did not use a native ligand due to lack of data in the protein data bank. Therefore, we explained the potential based on the docking score only. A conventional hydrogen bond has the main role to stabilize the interaction in the complexes. The result indicated that amino acids involved in the interaction are commonly similar. They bind on the region between 140–180 to indicate the same binding site.

Molecular interaction on spike protein showed that the ligand has a different binding site. Van der Waals interaction is the main type of interaction for all complexes. These data only explained for docking stability were supported by different interactions and also different interacting residues.

### 3.5. Theoretical Quantum Chemical Calculations

**Figure 6:**
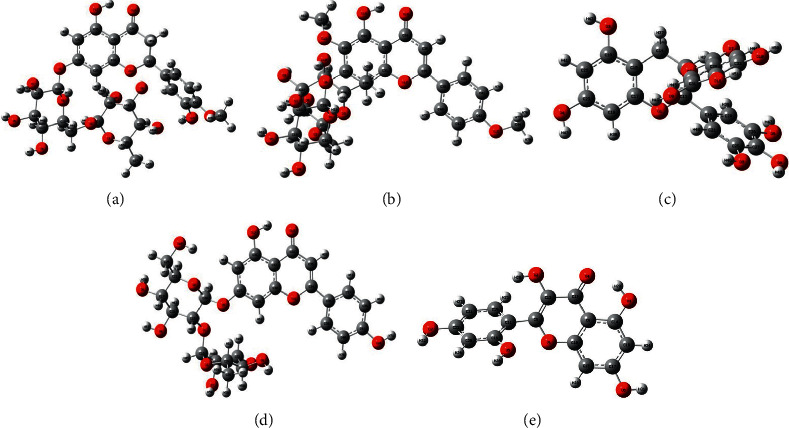
Optimized molecular structures of (a) hesperidin, (b) pectolinarin, (c) epigallocatechin gallate, (d) rhoifolin, and (e) morin, calculated by the DFT/B3LYP/6-31G level of theory.

The optimized molecular structures calculated at the DFT/B3LYP/6-31G level and numbering of the atoms of the selective best docking score of compounds are given in Figure 6, and energy with dipole moment values are presented in Table 3. The selective compounds showed dipole moments of 8.310, 8.441, 3.220, 4.761, and 7.630 Debye for hesperidin, pectolinarin, epigallocatechin gallate, rhoifolin, and morin, respectively. Also, all structures showed stable conformation, with a C1 symmetry and good structural cohesion revealing energy values of −2215.06062 a.u (−5815642.10 kJ/mol) for hesperidin, −2253.13700 a.u (−5915611.64 kJ/mol) for pectolinarin, −1676.10542 a.u (−4400615.11 kJ/mol) for epigallocatechin gallate, −2099.37177 a.u (−5511901.00 kJ/mol) for rhoifolin, and −1103.83213 a.u (−2898111.47 kJ/mol) for morin.  Table 4 shows global reactivity descriptor values of the best docking score of compounds at the gas phase.

**Figure 7:**
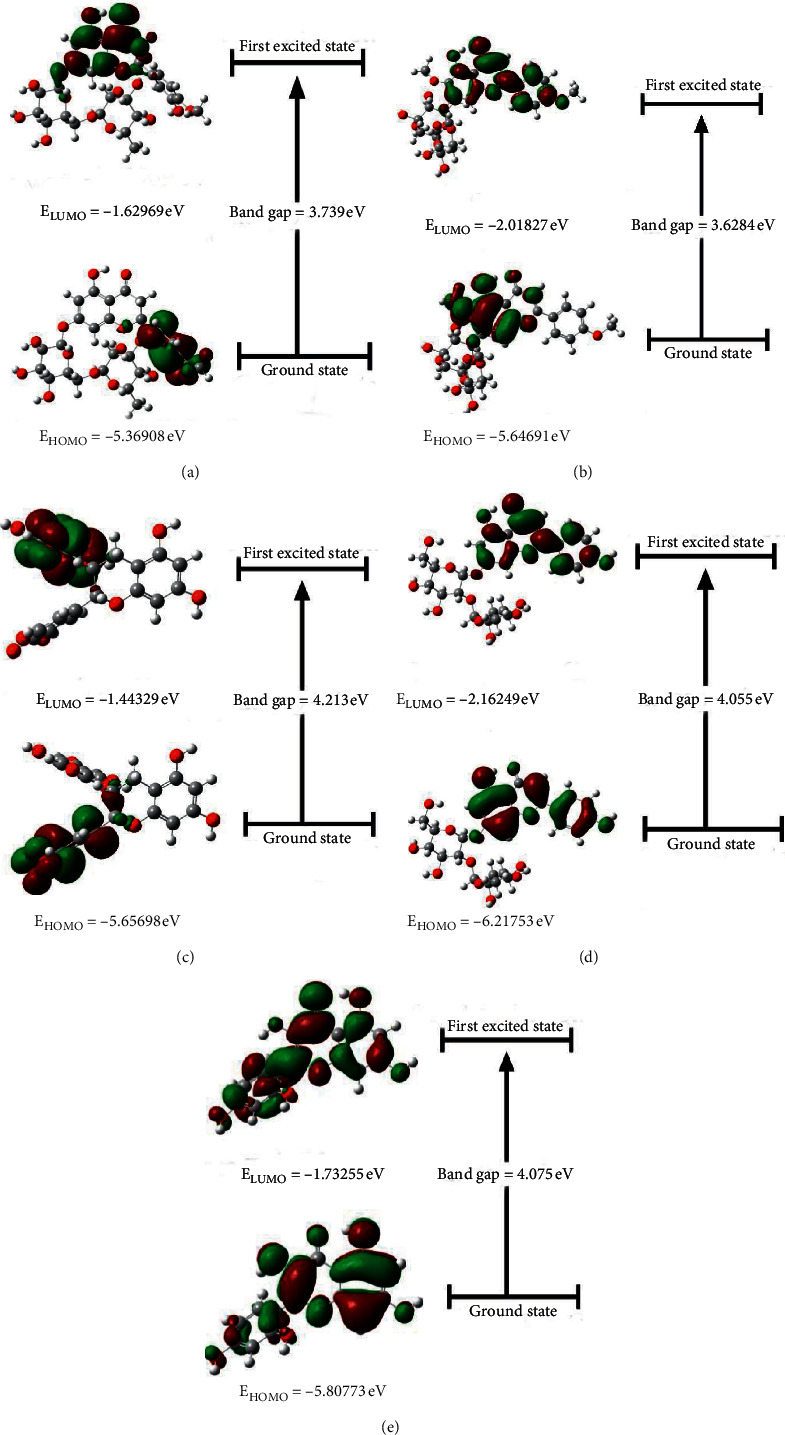
HOMO-LUMO energy values and bandgap of (a) hesperidin, (b) pectolinarin, (c) epigallocatechin gallate, (d) rhoifolin, and (e) morin, respectively, predicted by the DFT/B3LYP/6-31G basis set.

The quantum bonding features for hesperidin, pectolinarin, epigallocatechin gallate, rhoifolin, and morin are depicted by the HOMO and LUMO plot with bandgap, as shown in Figure 7, calculated by the DFT/B3LYP/6-31G level of theory in the gas phase.

**Figure 8:**
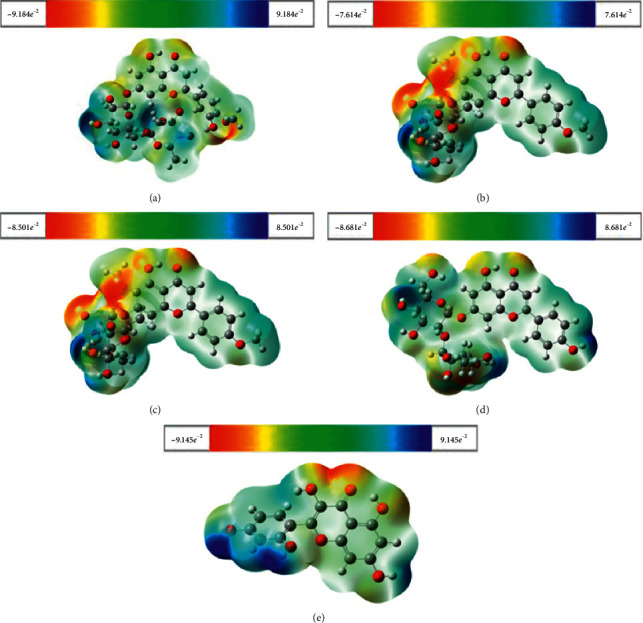
Calculated 3D surface map of electrostatic potential for (a) hesperidin, (b) pectolinarin, (c) epigallocatechin gallate, (d) rhoifolin, and (e) morin, respectively, in (a.u), the electron density isosurface being 0.0005 (a.u).

The MEPS map for the selective best docking score of compounds predicted by the DFT/B3LYP/6-31G method with 0.0005 isosurface value is shown in Figure 8 by using Gauss view 6.0.10 computer software. Different colors represent the different values of the electrostatic potential at the surface. Red color represents the maximum negative area, a favorable site for an electrophilic attack. Blue color indicates the maximum positive area, a favorable site for a nucleophilic attack, and green color represents the zero potential area. MEPS displays molecular size and shape, as well as positive, negative, and neutral electrostatic potential regions simultaneously in terms of color grading. The potential values for selected compounds such as hesperidin, pectolinarin, epigallocatechin gallate, rhoifolin, and morin range from −9.184*e*^−2^ a.u to +9.184*e*^−2^ a.u, −7.614*e*^−2^ a.u to +7.614*e*^−2^ a.u, −8.501*e*^−2^ a.u to +8.501*e*^−2^ a.u, −8.681*e*^−2^ a.u to +8.681*e*^−2^ a.u, and −9.145*e*^−2^ a.u to +9.145*e*^−2^ a.u, respectively.

### 3.6. Plants Containing Docking Compounds

The list of plants that have active compounds used as ligands is presented in Table 5. The table shows that citrus fruit have many active compounds, which are potential anti-SARS-CoV-2, including hesperidin, rhoifolin, nobiletin, tangeretin, and chalcone. The table shows that only pectolinarin, epigallocatechin gallate, myristicin, and eugenol have high bioavailability when administered orally.

## 4. Discussion

### 4.1. The Drug Likeness

Several different classes of bioactive molecules isolated from many plants have been shown to have antiviral activity [74, 75]. In determining that a compound has the potential as a drug, one of the methods is to follow the rule of five (Ro5). According to this rule, orally active drugs must not have more than one violation of established criteria [76]. Therefore, whether each docking compound met Lipinski's RO5 was checked. Some compounds that show violations towards RO5 are hesperidin (3), nabiximols (2), pectolinarin (3), epigallocatechin gallate (2), and rhoifolin (3) (Table 1). The rule is used for the evaluation of the drug-likeness, as well as a determination if any particular chemical compound possesses chemical and physical properties to be used as an active drug, which can be consumed orally in humans [46]. It also acts as a basis for predicting a high probability of success or failure of one compound with particular pharmacological or biological activity to be developed as a drug. This rule also suggests that if a compound shows two or more Ro5 violations, then it shows low solubility or permeability [77].

### 4.2. Validation of the Molecular Docking Process

To validate the results, M^pro^ was redocked. This redocking's binding site area was *x*: 7.808, *y*: 18.739, and *z*: 65.479 with a center grid box 24 × 24 × 24. The parameter of the validation method is RMSD (Root Mean Square Deviation). RMSD showed the degree of deviation from experimental ligand docking results to the crystallographic ligand at the same binding site. The higher the RMSD value, the greater the deviation, which indicates the higher prediction error of ligand-protein interactions [78]. Conversely, the smaller RMSD value obtained shows better conformation because the redocking ligand position is closer to the ligand position resulting from the crystallography [79]. The result indicated that the RMSD value obtained from the native ligand with the M^pro^ receptor was 1.281 Å, so it can be said that the method used for docking in this study is valid and can be used against tested ligands with the same binding site area. In addition to generating data in the form of RMSD values, in the validation stage, data were also obtained in the form of binding affinity values between ligands and receptors of −7.5 kcal/mol. There were also types of bonds that were formed between the native ligand and amino acid residues in proteins, such as hydrophobic interaction, hydrogen bond, and van der Waals interactions.

### 4.3. Molecular Docking

Dozens of proteins are coded by a coronavirus, some of which are involved in viral replication and entry into cells. Main protease (M^pro^/3CL^pro^) is a crucial enzyme for coronavirus replication [80], and surface Spike (S) glycoprotein (S protein) is an essential binding protein for the fusion of the virus and cellular membrane via cellular receptor angiotensin-converting enzyme 2 (ACE2) [81]. SARS-Cov-2 is easily transmitted because the S protein on the virus's surface binds very efficiently to ACE2 on the human cells' surface. Therefore, M^pro^ and S protein are ideal targets for drug design and development.

Efforts have been made globally to obtain vaccines or drugs for the prevention or treatment of COVID-19 infections. So far, remdesivir is the most promising COVID-19 drug, although the FDA has also approved the use of chloroquine and hydroxychloroquine. Coutard et al. [82] suggested finding an inhibitor for furin because the S protein sequence has a specific furin-like cleavage. Besides, some researchers have targeted M^pro^/3CL^pro^ for treating coronavirus infection [25, 83].

This study, which aimed at predicting the inhibition ability of compounds found in some plants against M^pro^ and S proteins, has revealed several results, showing that these compounds have a better docking pose than nelfinavir, chloroquine, and hydroxychloroquine sulfate (Table 2 and Figure 1). If the results are juxtaposed, the potential candidates to become drugs targeting S protein and M^pro^ were hesperidin, nabiximols, rhoifolin, pectolinarin, morin, epigallocatechin gallate, and herbacetin.

The glycoprotein spike (S protein) receptor does not have a target structure equipped with an inhibitor in the Protein Data Bank (PDB) because this receptor is a receptor that binds to the human ACE2 receptor (hACE2). The inhibition does not target the S protein receptor. Still, it occurs on the surface between the two receptors (S protein and hACE2), so that the binding site area is no longer on the spike glycoprotein receptors but between the two receptors [84, 85]. Therefore, the blind docking method was used for the S protein receptor in its molecular docking analysis.

There are three main criteria for carrying out molecular docking: bond intensity, molecular linkages, and bond characterization. Lead compounds have very small bond energies, hydrogen bonds, and van der Waals interactions and a good ADME profile [86]. Therefore, four ligands were selected as suitable lead compounds to inhibit the performance of the M^pro^ and in further studies based on the abovementioned criteria. These compounds are hesperidin, nabiximols, pectolinarin, and epigallocatechin gallate.

According to the research by Tahir ul Qamar et al. [25], the binding site area of M^pro^ is located on the active sites of Cys-145 and His-41. The ligands that bind to this receptor's active site can significantly inhibit the performance of the receptor. The ligand interactions that have the lowest binding affinity were hesperidin, pectolinarin, and epigallocatechin gallate, which indicated that the ligand was bound exactly to one of the active sites of the Cys-145 amino acid residue in the form of hydrogen bonds and van der Waals interactions. On the other hand, nabiximol did not bind to the active site of the enzyme.

The more the hydrogen bonds formed with the amino acid residue, the stronger the bonds. This causes the energy score to be lower, and the bonds will be more stable. Hydrogen bonds are interactions between hydrogen atoms (H), which are covalently bonded with atoms such as fluorine (F), nitrogen (N), and oxygen (O) [87]. In this study, each best ligand selected has a different number of hydrogen bonds and is located on a different amino acid residue. Hesperidin has four hydrogen bonds with M^pro^ at the amino acid residues Phe-A: 140, Glu-A: 166, Cys-A: 145, and Ser-A: 144. Nabiximol has three hydrogen bonds with Mpro, which resides at residues Thr-A: 190, Met-A: 165, and Asn-A: 142. Furthermore, the pectolinarin is hydrogen bonded with M^pro^ at the amino acid residues Glu-A: 166, His-A: 163, Cys-A: 145, and Ser-A: 144. Meanwhile, epigallocatechin gallate has three hydrogen bonds with M^pro^ in the residues Thr-A: 190, Met-A: 165, and Asn-A: 142.

Spike protein is considered a potential receptor target for discovering new types of drugs [84]. Spike proteins, both in the form of closed state (6VXX) and open state (6VYB), have amino acid residual bonds in the form of van der Waal's interactions, hydrogen bonds, and hydrophobic interactions. Hydrogen bonds occur in each ligand that binds to the S protein receptor. The hydrogen bonds are in the amino acid residues Asn-B: 1023, Ser-A: 1030, Thr-A: 1027, Gln-A: 762, Lys-A: 176, Ser-B: 1030, Arg-C: 1039, Asn-C: 1023, Gln-A: 762, and Lys-A: 776. Hydrophobic interactions avoid a liquid environment and tend to cluster in proteins' inner globular structure [88]. Hydrophobic interactions can be in the form of Pi-Sigma and Alkyl/Pi-Alkyl bonds [89]. This study shows that each ligand has hydrophobic interactions that can support receptor inhibition. As for the van der Waals bond, it contributes to the ligand to inhibit the target receptor because of the large number even though the strength of this interaction is not as strong as that of the hydrogen bond. Van der Waals bonds are relatively weak electric attractions due to induced or permanent polarity of molecules [90].

The results of the interaction between the S protein and the selected ligands show that there are unfavorable donor-donor bonds, which means that this bond shows the repulsive force between the two molecules. The formation of this bond can reduce the stability of other types of bonds so that it can affect the stability of the ligands that will be used as drug candidates [91]. The ligands with this type of bond are hesperidin and pectolinarin, located in the residue Arg-A: 1039 and Arg-B: 1039.

### 4.4. Theoretical Quantum Chemical Calculations

The highest occupied molecular orbitals (HOMO) characterize the electron-donating ability of a molecule, and the lowest unoccupied molecular orbitals (LUMO) determine the ability to accept an electron also known as frontier molecular orbitals (FMOs), which are essential to determine the way the molecule interacts with other species, electric and optical properties, kinetic stability, molecular reactivity, and chemical reactivity descriptors, as softness and hardness [92–94]. The bandgap between the HOMO and LUMO is very important in determining the chemical reactivity of the molecule. In terms of chemical hardness, the obtained HOMO-LUMO bandgap can give valuable information, where a large energy gap indicates hard and more stable molecules and a small energy gap indicates a soft and more reactive molecule. Among the five selected compounds, pectolinarin shows the lowest bandgap, suggesting that it is more reactive than other compounds. The chemical reactivity order of the three selected compounds was pectolinarin > hesperidin > rhoifolin > morin > epigallocatechin gallate.

The global reactivity descriptors such as hardness (*η*), softness (*S*), chemical potential (*µ*), electronegativity (*χ*), and electrophilicity index (*ω*), which are calculated from HOMO and LUMO energies, were obtained by the level of theory DFT/B3LYP/6-31G and incorporated in Table 4. Using Koopmans' theorem [95, 96], IA and EA values can be correlated with the frontier orbitals by the relation IA = −E_HOMO_ and EA = −E_LUMO_. Reactivity descriptors such as global hardness and global softness (*S*) are defined as *η* = (IA–EA)/2 and *S* = 1/*η*, chemical potential is described as *µ* = −*χ*, the absolute electronegativity (*χ*) is given by the relation *χ* = (IA + EA)/2, the electrophilicity (*ω*) can be calculated using the electronic chemical potential, and the chemical hardness is described as *ω* = *µ*^2^/2*η* [97–101].

The original basis for the concept of hardness (*η*) and softness (*S*) lies in observations made by inorganic chemists from the coordination chemistry and is related to a compound's reactivity. Soft ions/molecules are more polarizable species and more reactive since the electrons are farther from the nucleus. In contrast, hard ions/molecules are less polarizable and less reactive, since the electrons are closer to the nucleus. The chemical potential (*μ*) is a greatness that defines the flow of matter. In general, a system always tends to shift from greater chemical potential to lower chemical potential, since this is its most stable configuration. The greatness given as the negative of the chemical potential is the electronegativity (*χ*). For any system, the value *χ* is called the absolute electronegativity and is related to the power to attract electrons [102]. Another important descriptor is the electrophilicity index (*ω*), a global maximum reactivity index that measures the energy lowering due to charge transfer. The electrophilicity index allows classification of organic molecules as strong with *ω* > 1.5 eV, moderate with 0.8 < *ω* < 1.5 eV, and marginal electrophiles with *ω* < 0.8 eV [103].

Hesperidin has the lowest ionization potential value (IA = 5.369 eV), which indicates that it is the best electron donor. The calculated hardness values (*η*) for hesperidin (1.86 eV), pectolinarin (1.81 eV), epigallocatechin gallate (2.10 eV), rhoifolin (2.02 eV), and morin (2.03 eV) show that pectolinarin is the softer and more reactive one and epigallocatechin gallate is the harder and less reactive molecule, confirming the evidence obtained by the calculation of the bandgap. Comparing these hardness values with those calculated for other known alkaloids, such as liriodenine (*η* = 1.81) [104], annomontine (*η* = 1.94), and N-hidroxyannomontine (*η* = 1.69) [105], pectolinarin and hesperidin present values that classify them as soft molecules. The chemical potential *µ* (eV) measures the escaping tendency of an electron, and it can be associated with the molecular electronegativity [106]; then, as *µ* becomes more negative, it is more difficult to lose an electron but easier to gain one.

As shown in Table 4, rhoifolin is the least stable among all isolated compounds. Electronegativity (*χ*) represents the ability of molecules to attract electrons. The (*χ*) values displayed in Table 4 show that rhoifolin has higher electronegativity (4.190 eV) value than other isolated compounds. Electrophilicity (*ω*) gives an idea of the stabilization energy when the system gets saturated by electrons, which come from the external environment. This reactivity information shows that a molecule is capable of donating charges. The electrophilicity index above 1.5 for each structure reveals that the selective compounds have a significative attractive electron power.

The molecular electrostatic potential surface (MEPS) [107] is a 3D plot of the electrostatic potential for a respective molecule mapped onto the constant electron density surface. Over the years, MEPS was established as a great and effective interpretive tool for intermolecular interactions [107]. With the recent advances in computational technology, it is currently being applied to give detailed information for studies on chemical reactivity (as well as the biological recognition process and hydrogen bonding interaction), crystal behavior, molecular cluster, and zeolite even as the correlation and prediction of a wide range of macroscopic properties [108]. Besides that, due to the density functional theory contributions, the MEPS is rigorously defined in terms of the electron density, and it explicitly reflects opposing contributions from the nuclei and the electrons [108–110]. All selected compounds are suitable for electrophilic and nucleophilic attack. C=O and O-H regions of all selected isolated compounds are most probably involved in the electrophilic and nucleophilic processes, respectively.

From the abovementioned quantum chemical calculations, it can be seen that pectolinarin is configurationally more stable than other compounds with maximum dipole moment, suggesting better binding affinity. The FMOs analysis indicated that both HOMO and LUMO are bonding orbitals and comprise the aporphine portion for each structure; however, pectolinarin has a bandgap smaller than that calculated for the other molecules, indicating that this molecule is more reactive. The electrophilicity index above 1.5 for all structure reveals that the compounds have a significative attractive electron power, and the small hardness (*η*) for hesperidin (1.86 eV), pectolinarin (1.81 eV), epigallocatechin gallate (2.10 eV), rhoifolin (2.02 eV), and morin (2.03 eV) reflects high polarizability for each molecule, showing pectolinarin as the softer one and epigallocatechin gallate as the harder structure. The predicted MEPS figure revealed that the selected compounds' positive and negative regions were subjected to the nucleophilic and electrophilic attack of those compounds.

### 4.5. The Potential of Each Docking Compound

Some of the plants producing compounds which are docked with the target protein can be seen in Table 5. This table also contains information on the oral bioavailability of the compounds used as ligands in this analysis. However, only few compounds have high bioavailability when administered orally based on studies that have been conducted by several other researchers, i.e., pectolinarin, kaempferol, herbacetin, eugenol, and 6-shogaol. Of these, only pectolinarin does not meet Ro5. The low oral bioavailability has become a common problem in drug design, since it may pose failure to a new drug in clinical trials, even though the compounds have high efficacy in the in vitro and/or in vivo tests [111]. This may incur a problem faced by scientists in the pharmaceutical industry [112]. Therefore, a compound's oral bioavailability is essential to be taken into account when predicting the compound as a drug candidate. The oral availability of some compounds can be low if administered together with food. However, the oral availability of a compound can also be improved by various strategies [113, 114].

The major flavanone glycoside in the citrus peel is hesperidin [115]. Docking scores of this compound with S protein and M^pro^ were −10.4 and −8.3, respectively. Utomo et al. [116] have docked hesperidin against S protein (−9.6) and M^pro^ (−13.51). Chen et al. [117] revealed that the best hesperidin position against SARS-CoV-2 3C-like protease (3CL^pro^) was −10.1. Adem et al. [118] found that the ability of hesperidin was better than that of nelfinavir. Based on this finding, it can be seen that hesperidin has great potential to be a candidate for drugs, but its low oral bioavailability is a problem.

Cannabinoids are active compounds of *Cannabis sativa* and *C. indica*. The docking score of nabiximols (a combination of cannabidiol and tetrahydrocannabinol) against M^pro^ and S protein was −8 and −10.2, respectively. Besides being known as an antiherpes simplex virus [28], this compound also has anti-inflammatory activity [119]. However, some research studies show that this compound can increase the virus's pathogenesis to the host [119–121].

The docking results using rhoifolin as a ligand were −9.5 and −8.2 for S protein and M^pro^, respectively. Rhoifolin is a flavone that was first discovered in the fresh leaves of *Rhus succedanea* in 1952 [122]. Besides, this compound was also found in *Citrus grandis* [123]. The result of rhoifolin docking on S protein was −9.5 and M^pro^ was −8.2. The rhoifolin binding score for SARS-CoV 3CL^pro^ shows a value of −9.565 [31].

The induced-fit docking result of pectolinarin against SARS-CoV 3CL^pro^ was −8.054 [31]. In this study, the best pose between pectolinarin and S protein was −9.8 and −8.2 with M^pro^. Pectolinarin can be found in plume thistles (*Cirsium* spp). The morin docking result by Jo et al. [31] against SARS-CoV 3CL^pro^ was −8,930. The best docking scores of morin against S protein and M^pro^ were −8.8 and −7.8, respectively. Almond, old fustic, and guava contain a high quantity of this compound.

Kaempferol can be found in spinach and kale. The best position of kaempferol against S protein was −8.5 and −7.8 against M^pro^, while −8,526 was the best binding position of this compound against SARS-CoV 3CL^pro^ [31]. Ro5 calculation results show that this compound has a high potential to be used as a drug. Some researchers have previously stated that its oral bioavailability varies from low to good. Besides having been reported to have the ability as an antiviral, this compound also shows immunomodulatory and anti-inflammatory activities [124, 125].

Epigallocatechin gallate is found in high quantity in tea (*Camellia sinensis*), especially in the form of green tea. The best binding position of this compound against S protein was −9.8 and against M^pro^ was −7.8. It has been reported previously that this compound was able to inhibit the proteolytic activity of SARS-CoV 3CL^pro^ [126]. Although it does not meet the Ro5 and its oral availability is low, it has immunomodulatory and anti-inflammatory activities [127, 128].

Herbacetin, which can be found in *Rhodiola* sp. (golden root), has antiviral activity against vesicular stomatitis virus (VSV) and a prototype of negative-strand RNA virus such as rabies and influenza viruses [129]. The best binding pose of this compound against SARS-3CL^pro^ was −9.263, as reported by Jo et al. [31], while in this study, the binding score of −8.3 against S protein and −7.2 against M^pro^ were obtained. They also stated that herbacetin might act as a MERS-CoV 3CL^pro^ inhibitor. Herbacetin is a very potential candidate as an anti-SARS-CoV-2 because it meets Ro5 and has also been reported to have good oral bioavailability. Besides, this compound also has anti-inflammatory activity [130].

Two compounds found in Pangi leaves, bis (3, 5, 5-trimethylhexyl) phthalate and ethyl cholate, have the potential to be developed as anti-SARS-CoV-2 drugs, due to their good binding affinity with M^pro^ and S protein and also because they meet the Ro5. Although there is no prior information about their oral availability, both compounds were reported to inhibit HIV-1 protease in silico.

Other compounds such as nobiletin, tangeretin, chalcone, 6-gingerol, myristicin, eugenol, and 6-shogaol have a fairly good binding affinity with M^pro^ and S protein and meet RO5 criteria. These compounds, despite their low oral availability, have immunomodulatory and anti-inflammatory activities [40, 131–138].

## 5. Conclusions

Our study revealed that natural compounds hesperidin, nabiximols, pectolinarin, epigallocatechin gallate, and rhoifolin had better binding free energies with M^pro^ and S protein of SARS-CoV-2. Although the results of molecular docking of kaempferol, herbacetin, eugenol, and 6-shogaol are not as good as those compounds, they have good oral availability and also meet Ro5 criteria. These compounds have potential as antiviral phytochemicals that may inhibit the replication of the virus. These results are only preliminary screening to facilitate subsequent tests starting from in vitro and in vivo (in animal models or human clinical trials).

## Figures and Tables

**Table 1:** Lipinski's rule of five (RO5) of SARS-CoV-2 M^pro^/3CL^pro^ and S protein potential inhibitors.

Compounds	Molecular formula	Properties					
		Molecular weight (<500 g/mol)	Log*P* (<5)	H-bond donor (<5)	H-bond acceptor (<10)	Violations	Meet RO5 criteria
Nelfinavir	C_32_H_45_N_3_O_4_S	567.78	4.41	4	5	1	Yes
Chloroquine	C_18_H_26_ClN_3_	319.87	4.15	1	2	0	Yes
Hydroxy-chloroquine sulfate	C_18_H_28_ClNO_5_S	439.95	2.13	4	7	0	Yes
Hesperidin	C_28_H_34_O_15_	610.56	−1.06	8	15	3	No
Nabiximols	C_42_H_60_O_4_	628.92	9.12	3	4	2	No
Pectolinarin	C_29_H_34_O_15_	622.57	−0.09	7	15	3	No
Epigallocatechin gallate	C_22_H_18_O_11_	458.37	0.95	8	11	2	No
Rhoifolin	C_27_H_30_O_14_	578.52	−0.81	8	14	3	No
Morin	C_15_H_10_O_7_	302.24	1.2	5	7	0	Yes
Kaempferol	C_15_H_10_O_6_	286.24	1.58	4	6	0	Yes
Herbacetin	C_15_H_10_O_7_	302.24	1.33	5	7	0	Yes
Ethyl cholate	C_26_H_44_O_5_	436.62	3.5	3	5	0	Yes
Quinine	C_20_H_24_N_2_O_2_	324.42	2.81	1	4	0	Yes
Nobiletin	C_21_H_22_O_8_	402.39	3.02	0	8	0	Yes
Tangeretin	C_20_H_20_O_7_	372.37	3.02	0	7	0	Yes
Chalcone	C_15_H_12_O	402.39	3.30	0	1	0	Yes
6-Gingerol	C_17_H_26_O_4_	294.38	3.02	2	4	0	Yes
Bis (3, 5, 5-trimethylhexyl) phthalate	C_26_H_42_O_4_	418.61	6.47	0	4	1	Yes
Myristicin	C_11_H_12_O_3_	192.21	2.49	0	3	0	Yes
Eugenol	C_10_H_12_O_2_	164.20	2.25	1	2	0	Yes
6-Shogaol	C_17_H_24_O_3_	176.37	3.76	1	0	0	Yes

**Table 2:** Molecular docking analysis of several plant compounds against S protein (6VXX) and M^pro^ (6LU7).

Ligands	PubChem CID	Binding free energy (kcal/mol)	
		6VXX	6LU7
Nelfinavir	64143	−8.8	−8.2
Hydroxychloroquine sulfate	12947	−7.3	−6.6
Chloroquine	2719	−6.1	−5.3
Hesperidin	10621	−10.4	−8.3
Nabiximols	9852188	−10.2	−8.0
Pectolinarin	168849	−9.8	−8.2
Epigallocatechin gallate	65064	−9.8	−7.8
Rhoifolin	5282150	−9.5	−8.2
Morin	5281670	−8.8	−7.8
Kaempferol	5280863	−8.5	−7.8
Herbacetin	5280544	−8.3	−7.2
Ethyl cholate	6452096	−8.1	−6.7
Nobiletin	72344	−8.1	−6.4
Tangeretin	68077	−7.9	−6.5
Chalcone	637760	−7.5	−6.2
Quinine	3034034	−7.5	−6.9
6-Gingerol	442793	−6.3	−5.8
Bis (3, 5, 5-trimethylhexyl) phthalate	34277	−6.1	−5.6
Myristicin	4276	−6.1	−5.3
Eugenol	3314	−6.1	−5.4
6-Shogaol	5281794	−5.5	−5.8

**Table 3:** Optimized energy of the best docking score of compounds with a dipole moment.

Name	Energy (a.u)	Dipole moment (Debye)
Hesperidin	−2215.06062	8.310
Pectolinarin	−2253.13700	8.441
Epigallocatechin gallate	−1676.10542	3.220
Rhoifolin	−2099.37177	4.761
Morin	−1103.83213	7.630

**Table 4:** Global reactivity descriptor values of the best docking score of the compounds at the gas phase.

Name	IP (eV)	EA (eV)	*η*	*S*	*μ*	*χ*	*ω*
Hesperidin	5.36908	1.62969	1.86970	0.53485	−3.49939	3.49939	6.12285
Pectolinarin	5.64691	2.01827	1.81432	0.55117	−3.83259	3.83259	7.34437
Epigallocatechin gallate	5.65698	1.44329	2.10684	0.47464	−3.55014	3.55014	6.30173
Rhoifolin	6.21753	2.16249	2.02752	0.49321	−4.19001	4.19001	8.77810
Morin	5.80773	1.73255	2.03759	0.49078	−3.77014	3.77014	7.10698

IP = ionisation potential; EA = electron affinity; *η* = global hardness; *S* = global softness; *μ* = chemical potential; *χ* = electronegativity; *ω* = electrophilicity index.

**Table 5:** List of plants that have active compounds used as ligands and their bioavailability.

Compounds	Oral bioavailability	Sources
Hesperidin	Low [55]	Citrus fruit (*Citrus* spp.), peppermint (*Mentha* spp.), yellow toadflax (*Linaria vulgaris*)
Nabiximols	Low [56, 57]	Marijuana (*Cannabis* spp.)
Pectolinarin	Low to good [58, 59]	Plume thistles (*Cirsium* spp.), yellow toadflax (*Linaria vulgaris*)
Epigallocatechin gallate	Low [60, 61]	Tea (*Camellia sinensis*) (green tea), the skin of apple (*Malus domestica*), plum (*Prunus domestica*), onion (*Allium cepa*), hazelnut (*Corylus avellana*)
Rhoifolin	Low [62]	Rhus plant (*Rhus succedanea*), bitter orange (*Citrus aurantium*), bergamot (*Citrus bergamia*), grapefruit (*Citrus paradisi*), lemon (*Citrus limon*), lablab beans (*Lablab purpureus*), tomato (*Lycopersicon esculentum*), artichoke (*Cynara scolymus*), bananas (Musa spp.), grapes (*Vitis vinifera*)
Morin	Low [63]	Osage orange (*Maclura pomifera*), almond (*Prunus dulcis*), old fustic (*Chlorophora tinctoria*), guava (*Psidium guajava*)
Kaempferol	Low to good [64, 65]	Kale (*Brassica oleracea* var. *sabellica*), beans (*Phaseolus vulgaris*), tea (*Camellia sinensis*), spinach (*Spinacia oleracea*), broccoli (*Brassica oleracea* var. *Italica*)
Herbacetin	Good [65]	Golden root (*Rhodiola* spp.), gossypium (*Gossypium hirsutum*), common horsetail (*Equisetum arvense*), common boneset (*Eupatorium perfoliatum*)
Ethyl cholate	N/A	Leaf of football fruit/keluak (*Pangium edule*)
Nobiletin	Low [66]	Citrus fruit (*Citrus* spp.)
Tangeretin	Low [67]	Citrus fruit (*Citrus* spp.)
Chalcone	Low [68]	Citrus fruit (*Citrus* spp.)
6-Gingerol	Low [69]	Fresh ginger (*Zingiber officinale*)
Bis (3, 5, 5-trimethylhexyl) phthalate	N/A	Leaf of football fruit/keluak (*Pangium edule*)
Myristicin	N/A	Nutmeg (*Myristica fragrans*)
Eugenol	Good [70, 71]	Clove (*Syzygium aromaticum*)
6-Shogaol	Low to good [72, 73]	Ginger (*Zingiber officinale*)

## Data Availability

The data related to this article are available from the corresponding author upon request.
